# Anthrax Susceptibility: Human Genetic Polymorphisms Modulating *ANTXR2* Expression

**DOI:** 10.3390/toxins8010001

**Published:** 2015-12-22

**Authors:** Zhang Zhang, Yan Zhang, Minglei Shi, Bingyu Ye, Wenlong Shen, Ping Li, Lingyue Xing, Xiaopeng Zhang, Lihua Hou, Junjie Xu, Zhihu Zhao, Wei Chen

**Affiliations:** Beijing Institute of Biotechnology, No. 20, Dongdajie str., Fengtai District, Beijing 100071, China; zzhnng@yeah.net (Z.Z.); zany1983@163.com (Y.Z.); shiml79@126.com (M.S.); byy0804@gmail.com (B.Y.); shenwl1988@gmail.com (W.S.); lipingtry@163.com (P.L.); xinglyinfo@163.com (L.X.); zxp8565@aliyun.com (X.Z.); houlihua@sina.com (L.H.)

**Keywords:** *ANTXR2*, anthrax toxin, gene transcription, genetic polymorphism, gene structure

## Abstract

Anthrax toxin causes anthrax pathogenesis and expression levels of *ANTXR2* (anthrax toxin receptor 2) are strongly correlated with anthrax toxin susceptibility. Previous studies found that *ANTXR2* transcript abundance varies considerably in individuals of different ethnic/geographical groups, but no eQTLs (expression quantitative trait loci) have been identified. By using 3C (chromatin conformation capture), CRISPR-mediated genomic deletion and dual-luciferase reporter assay, gene loci containing cis-regulatory elements of *ANTXR2* were localized. Two SNPs (single nucleotide polymorphism) at the conserved CREB-binding motif, rs13140055 and rs80314910 in the promoter region of the gene, modulating *ANTXR2* promoter activity were identified. Combining these two regulatory SNPs with a previously reported SNP, rs12647691, for the first time, a statistically significant correlation between human genetic variations and anthrax toxin sensitivity was observed. These findings further our understanding of human variability in *ANTXR2* expression and anthrax toxin susceptibility.

## 1. Introduction

Anthrax toxin, consisting of three Bacillus anthracis-produced virulence factors, PA (protective antigen), LF (lethal factor) and EF (edema factor), gains cytosolic access and disturbs host defense signaling pathways to enable anthrax spore survival and germination. Strains lacking the anthrax toxin are avirulent and the toxin itself can lead to toxemia, serious organ damage, or death of infected hosts [1,2,3]. PA, LF and EF can pair to form LT (lethal toxin, composed of PA + LF) and ET (edema toxin, composed of PA + EF). PA is the cellular-binding moiety, and LF and EF are catalytic moieties of the toxins. To deliver effector LF and EF into cytosol, PA binds to the cell surface receptors ANTXR2 and ANTXR1. After being processed by furin proteinase, LF/EF bind to PA oligomers to form toxin-receptor complex. The uptake of toxin complex is then triggered through a receptor-mediated endocytic routine. The acidification of the endosomes induces LF/EF translocated into cytosol through the PA oligomer protein conducting channel to exert their cytotoxic effects. As for the important role of PA in cellular binding, toxin complex formation and internalization, many antibodies against PA, especially those blocking toxin binding to its receptors, are proven to have a highly protective effect in anthrax infection. Due to the rapid course of anthrax and the continuing effects of the toxins, besides antibiotics, antibodies against PA are indispensable for anthrax infection therapies. Abthrax, the first FDA (Food and Drug Administration)-approved human monoclonal PA-neutralizing antibody available since 2012, functions in this way [2]. The internalization of anthrax toxin depends on several host membrane proteins, such as ANTXR1, ANTXR2, ARAP3, LRP6, COP1, Cathepsin B, GRP78, Calpain, and TCP-1, among others [4,5,6,7,8,9,10,11]. Interrupting the expression of the encoding genes could enhance host resistance to anthrax toxin. Among these cellular genes exploited by pathogens, *ANTXR2* is the prominent determinant, and inactivation of the gene generates the strongest protective effect, rendering the host completely resistant to anthrax toxin and the bacterium [12,13,14,15].

One study carried by Martchenko *et al.* [16] found that different ethnic/geographic groups showed remarkable variation in terms of relative sensitivity levels to the protective antigen-mediated toxin which has been widely used as a surrogate in investigations of anthrax toxin entry mechanisms. This susceptibility trait variation is heritable and correlates strongly with *ANTXR2* transcript abundance, which also varies considerably in populations. However, transcriptional regulation of *ANTXR2* is largely unknown and no eQTLs have been identified yet. Since several studies have demonstrated that genetic variation in regulatory elements can affect transcription levels of cognate genes [17,18], it is reasonable to suppose the presence of such polymorphisms, especially those at transcription factor binding sites (TFBS) adjacent to promoter or enhancer elements, might modulate *ANTXR2* expression and consequently affect anthrax toxin uptake and cellular susceptibility.

To test this hypothesis, we focused on SNPs (single nucleotide polymorphism) located in TFBS in cis-acting elements of *ANTXR2*, especially in CREB (c-AMP response element binding protein) motifs, as this is the only transcription factor reported to be involved in the process. By using 3C and CRISPR-mediated (CRISPR, clustered regularly interspaced short palindromic repeat) sequences in *in situ* genomic deletion, regions containing regulatory elements, including promoter and enhancer elements, were identified, and two SNPs located in CREB-binding motifs were identified with a combinatorial influence on promoter activity in dual-luciferase reporter assay. Experiments using episomes suggested that promoter variants with G at rs13140055 and CTT at rs80314910 might result in elevated *ANTXR2* expression. These two SNPs demonstrably affected anthrax receptor abundance and the SNP rs12647691 experimentally altered the binding affinity between toxin and receptor in the study by Martchenko *et al.* We show that these SNP genotypes are statistically associated with anthrax toxin susceptibility.

## 2. Results

**Figure 1:**
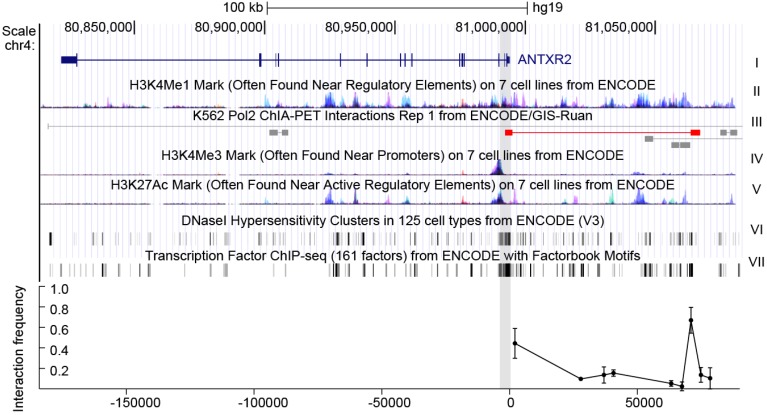
The gene context and structure of *ANTXR2*. **Top**: genomic context of *ANTXR2* and upstream regions. The first track represents *ANTXR2* gene loci; the H3K4Me1 mark in track II is an indicator of the enhancer, the H3K4Me3 mark and H3K27Ac mark in track IV and V are indicators of the promoter; the DNaseI hypersensitivity clusters mark in track VI is an indicator of active chromatin; the transcription factor ChIP-seq (chromatin immunoprecipitation sequencing) mark in track VII is an indicator of transcriptional active chromatin; in track III, long-range interactions in ChIA-PET data similar to interactions detected in the present study are marked red. Gray shading indicates putative promoter and anchor regions used in 3C assays; **Bottom**: interaction frequency between fragments digested by restriction enzyme and cognate promoter in cross-linked HEK293 cells. The error bars represent biological replicates (*n* = 3).

We postulated that genetic polymorphisms modulating *ANTXR2* expression might be located in regulatory elements. To identify putative *ANTXR2* regulatory motifs, we searched relevant data from the ENCODE (encyclopedia of DNA elements) project database [19]. A fragment of 2 kb (chr4:80993755-80995687) around the transcription start site (TSS), characterized by indicators characteristic of promoters, including H3K4Me3 (trimethylation of histone H3 at lysine 4) and H3K27Ac (acetylation of histone H3 at lysine 27) signals, DNase I (deoxyribonuclease I) hypersensitivity and high affinity for transcription factors, was predicted as the promoter of *ANTXR2* (Figure 1) [20,21,22].

There are several additional H3K4 methylation peaks upstream of ANTRX2, indicating that enhancers might also play roles in ANTRX2 regulation. To extend the survey of cis-acting elements for SNPs and TFBSs, we included enhancer elements, especially the distal one. A great deal of evidence suggests that distal enhancers play an important role in transcriptional activation via long-range interactions [17,18]. These interactions could be detected by chromatin conformation capture assays such as 3C, 4C (circular chromosome conformation capture), Hi-C, ChIA-PET (Chromatin Interaction Analysis by Paired-End Tag Sequencing) and others. According to chromatin state features and ChIA-PET data from the ENCODE project, the interaction between the *ANTXR2* promoter and fragments across 80 kb of the TSS upstream had been evaluated in HEK293 cells and THP-1 cells by 3C assays. The greatest interaction between the promoter and a putative enhancer characterized by a H3K4Me1 (monomethylation of histone H3 at lysine 4) peak [21] was observed about 70 kb upstream of the TSS (Figure 1 bottom, data for THP-1 can be found in Figure S1 in supplementary data). These data indicate that the putative promoter as well as the distal enhancer may be responsible for expression control of *ANTXR2* in these cell lines.

Conserved TFBS analysis via rVISTA 2.0 [23] suggested the region containing the putative promoter harbors 424 conserved TFBSs, while ChIP-seq data from the ENCODE project indicated that approximately 131 different TFs bind to this locus in tested cell lines. The putative enhancer has 509 conserved TFBSs, with 23 different TFs binding to this region, as revealed by ChIP-seq. Among the hundreds of TFs which potentially bind at predicted regulatory regions, we chose to test the effects of genetic variation within the CREB-binding motif. To our knowledge, CREB is the only transcription factor reported to regulate *ANTXR2*. According to previous studies, ET has calmodulin-dependent adenylate cyclase activity and raises *ANTXR2* expression in target cells and the up-regulation process could be interrupted by repressing phosphorylation of CREB. This interesting phenomenon suggested edema toxin enhances toxin sensitivity of host cells after initial infection by inducing *ANTXR2*.

Because *ANTXR2* recruits CREB for transcription, SNPs in these TFBS might not only affect gene transcription in physiological conditions but also alter host susceptibility to anthrax infection [24,25,26,27]. Additionally, there are two SNPs, rs13140055 and rs80314910, located within neighboring CREB-binding motifs in the predicted promoter. So we focused our study on the CREB-binding sites containing SNPs, and tested whether these SNPs would affect *ANTXR2* expression and cell susceptibility to anthrax toxins.

**Figure 2:**
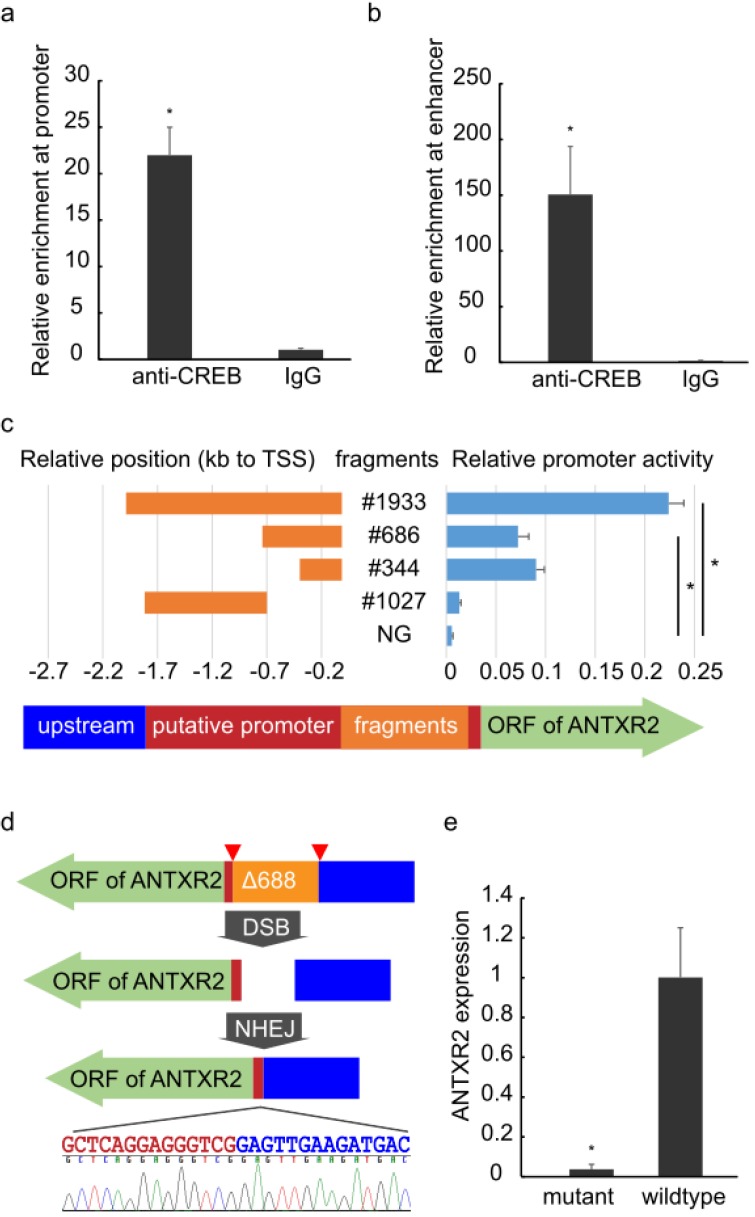
Identification of regulatory regions.ChIP-qPCR analysis of the promoter (**a**) and enhancer (**b**) areas of *ANTXR2* was performed using anti-CREB1 antibody and IgG showing enrichment of the regions containing the SNP to be analyzed or a long-range interacting element. The error bars represent technical replicates (*n* = 4); (**c**) **Left**: Orange bars indicate relative positions of fragments within the region of the putative promoter #1933 (chr4: 80993755-80995687), #686 (chr4: 80993755-80994440), #344 (chr4: 80993755-80994098), #1027 (chr4: 80994440-80995466); the arrow indicates the direction of transcription and open reading frames (green arrow), putative promoter regions (red) and upstream (blue); **Right**: Blue bars show the promoter activity of corresponding fragments measured by dual-luciferase reporter assays. The promoter activity of #1933 is significantly higher than in other groups, #686 and #344 are significantly higher than #1027 and NG. NG: negative control (empty pGL-3 Basic plasmid). The error bars represent biological replicates (*n* = 4); (**d**) Schematics of CRISPR design: Plasmids expressing two guide RNAs and Cas9 were co-transfected into HEK293 cells. Pairs of guide RNA (red triangles)-directed Cas9 nuclease could create DNA double-stranded breaks (DSBs) at the ends of fragment #686 (orange). Subsequently, the non-homologous end joining (NHEJ) pathway would repair the DSBs, generating a mutant whose guide RNA flanking fragment was seamlessly removed, without any selective tag insertion, and the large deletion was verified by Sanger sequencing. Color-labeled sequencing results illustrate the seamless deletion; (**e**) The abundance of *ANTXR2* transcripts in mutant and wild-type HEK293 cells measured by RT-qPCR. The error bars represent technical replicates (*n* = 4). *p*-value < 0.05 was considered statistically significant and mark with asterisk.

A ChIP-qPCR assay was then performed to detect CREB binding at the *ANTXR2* promoter, especially in the region containing these two SNPs. An enrichment of CREB binding to the SNP harboring region as well as the putative enhancer was confirmed in HEK293 (Figure 2a,b). The CREB binding to these elements was cross-validated by ChIP-seq data from the ENCODE project in several other cell lines, including GM12878, hESC, K562, HepG2 and ECC, suggesting that CREB binding to these regions is not cell-type specific. We treated HEK293 and THP-1 cells with Forskolin, an agonist of adenylate cyclase, but did not observe significant up-regulation of *ANTXR2*, as previously reported [24]. We reasoned that co-factors are needed for CREB regulation of *ANTXR2*, and further work should be done to elucidate transcriptional regulation on *ANTXR2*.

To evaluate any regulatory impact of these two SNPs, several overlapping fragments were cloned into the pGL3-Basic vector to examine their promoting activity by using dual-luciferase reporter assay (Figure 2c left). The fragment #686 (686 bp) displayed remarkable promoter activity experimentally, implicating this region as critical to initiate transcription. If the fragment was extended to encompass elements further 5′ upstream to −1933 bp, additional activity could be observed. However, the extended region (#1027) alone exhibited no activity at all, suggesting that #686 contains the promoter and initiates transcription of *ANTXR2* while upstream regions further enhance the process. After trimming the #686 component to 344 bp (which still contains the SNPs and the CREB motifs), similar promoter activity was still observed, indicative of a likely core promoter (Figure 2c right).

CRISPR experiments were performed to confirm the promoter function of #686 via genomic deletions *in vivo*. After several rounds of screening from 40 clones, an anticipated homozygote (HEK293∆688, 688 bp deletion) was isolated (Figure S2) and verified by Sanger sequencing (Figure 2d). RT-qPCR revealed that the expression level of *ANTXR2* in HEK293∆688 was considerably diminished (Figure 2e), indicating that *ANTXR2* expression is dependent on this region containing the two SNPs previously mentioned.

**Figure 3:**
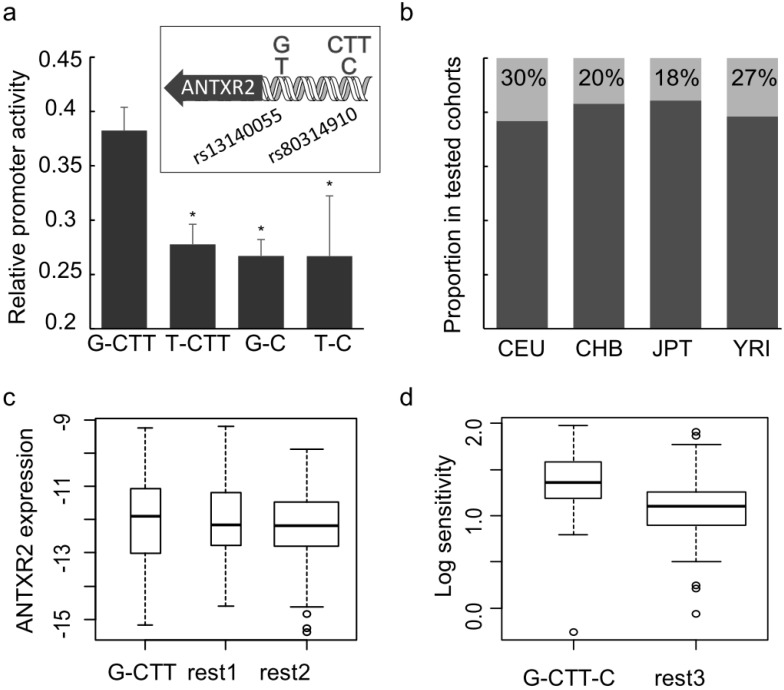
Effects and distribution of SNPs rs13140055 (G or T) and rs80314910 (CTT or C). (**a**) The effects of SNPs rs13140055 and rs80314910 on promoter activity were measured by dual-luciferase reporter assays. G-CTT group is significantly higher than other groups. The error bars represent biological replicates (*n* = 4); (**b**) Proportion of SNPs in four HapMap ethnicities. Areas of light gray indicates G-CTT homozygotes; (**c**) *ANTXR2* expression in G-CTT homozygotes is marginally higher than in other genotypes (but not statistically significant). “rest1”: population whose genotypes are homozygous G at rs13140055 or homozygous for CTT at rs80314910, “rest2”: population not homozygous for G and CTT for both SNPs; (**d**) The Log sensitivity of homozygotes of G-CTT and C at rs13140055, rs80314910 and rs12647691 shows higher sensitivity than other genotypes with statistical significance (*p* = 0.0167). “rest3”: population whose genotypes are not homozygous for G-CTT and not C-homozygotes at rs12647691. *p*-value < 0.05 was considered statistically significant and mark with asterisk.

Next, the influence of these two genetic variants on *ANTXR2* promoter activity was quantified using dual-luciferase reporter assay, and an interesting observation was that higher transcription levels were only observed when the promoter region contained both the G-allele at rs13140055 and CTT-allele at rs80314910 (Figure 3a). We noted that other genotype combinations did not exhibit significant differences, which suggested disrupting either motif is sufficient to impair *ANTXR2* expression.

The 1000 Genomes project surveyed both SNPs in the same cohort as HapMap, where *ANTXR2* expression levels and sensitivity to anthrax toxin were detected in the study of Martchenko *et al.* [16] The data show that the proportion of homozygous G-CTT genotypes is approximately one quarter in all cell populations (Figure 3b). We merged the previous data of *ANTXR2* transcript abundance with corresponding genotype records and did not observe a statistically significant association (Figure 3c). This result is not unexpected since the regulation of *ANTXR2* is a multi-determinant mechanism and the tested cell populations are genetically complicated, with the effect of two SNPs identified here perhaps obscured by effects of other regulatory determinants. However, after taking into account rs12647691 (the reported variant in the *ANTXR2* coding region altering toxin uptake, with the C-allele resulting in greater toxin affinity than the G-allele) for genotype-sensitivity analysis, it was found that homozygotes of G-CTT-C at rs13140055, rs80314910 and rs12647691 are more sensitive than heterozygotes (*p* = 0.0167) (Figure 3d). This suggests that multiple determinants are involved in susceptibility differences to anthrax toxin in human populations, and the two genetic variants identified here contribute to this.

To determine the effect of genetic variation on the binding of transcriptional factors, EMSA (Electrophoretic Mobility Shift Assay) and Supershift with anti-CREB antibody were performed (Figure S3). The results indicated that both SNPs, rs13140055 and rs80314910, are located at the CREB-binding motif. The probe standing for CTT in rs80314910 had better affinity to CREB, which is consistent with the promoter activity assay (Figure 3a). However, probes representing rs13140055 variation did not appear to show a significant difference in CREB binding, which implied other mechanisms, rather than affecting CREB binding, might determine the SNP’s effect on promoter activity.

## 3. Discussion

The strong correlation between *ANTXR2* expression and host susceptibility to anthrax toxin has been demonstrated in many cells and animal models; recently, it has been confirmed in HapMap cells using population-based statistical analysis [2,12,13,14,15,16]. The only reported genetic variation altering toxin sensitivity is rs12647691, which affects the binding affinity between anthrax toxin and its receptor via a non-synonymous SNP. In this study, for the first time, the promoter of *ANTXR2* was characterized by dual-luciferase assay in episomes and verified by CRISPR/Cas9-mediated genomic deletion *in vivo*, and two genetic variations occupying CREB binding sites were found to affect promoter activity synergistically. Also, a region of 3550 bp in length which is 70 kb upstream to TSS was also confirmed to be bound by CREB and spatially co-localized to the promoter region, suggesting that there is additional transcriptional regulation governing *ANTXR2* expression. It should be interesting to further investigate whether there are any polymorphisms in this region accounting for *ANTXR2* expression variation. Considering the range of population variation in *ANTXR2* expression levels, the present findings augment understanding of the regulatory mechanisms involved. Additional genetic variation in these regions may well be detected in terms of influence on *ANTXR2* expression, raising the prospect that a panel could be constructed and used for anthrax toxin susceptibility prediction.

In the analysis of *ANTXR2* expression or toxin sensitivity correlated to populations carrying different genotypes, as shown in Figure 3c,d, we set up three groups rather than many groups for every genotype. We noticed that heterozygotes are the majority in groups of rest1, rest2 and rest3, which is likely to confound the analysis. It is because the numbers of individuals carrying certain genotypes are significantly different. For example, only five individual cells have C/C at rs80314910 in the available data, and the genotypes at rs13140055 and rs12647691 in these five cells are G/G and C/C. Therefore, based on the experiment results showed in Figure 3a, with the presumption that homozygotes have a clearer phenotype than heterozygotes, we focused on G-CTT or G-CTT-C homozygotes and combined other genotypes into three groups to ensure enough samples in each group for statistical analysis.

Interestingly, two SNPs examined in this study have shown their combinatorial effect on *ANTXR2* promoter activity, while it is difficult to discern in GWASs (genome wide association study) since the tested populations do not provide sufficient cohorts of each genotype. Given the complex genetic make-up of cell populations, it is not unusual for an association which has been experimentally demonstrated *in vitro* to not be observed statistically in populations. However, when three SNPs (one modulating toxin affinity and two others coordinately altering expression levels of the receptor) were analyzed for toxin susceptibility, a statistically significant association could be observed. This result supports the influence of genetic variants found here with the notion that transcription of *ANTXR2* is a multi-factorial mechanism, and confirms the role of genetic variation found by Martchenko *et al.* [16].

Among the SNPs in the regulatory region of *ANTXR2*, we focused on these two SNPs because their positions were predicted as CREB-binding sites, and ChIP-qPCR and EMSA confirmed the CREB involvement. Both SNPs affect promoter activity in dual-luciferase reporter assay but Supershift revealed that only variation at rs80314910 affect CREB binding (Figure S3b). It suggested that, although CREB binds at rs13140055, it is not an essential determinant in having an effect on promoter activity. rVISTA 2.0 analysis showed that this SNP is also located at some other TFs’ binding motifs. Further research about the transcriptional mechanism might provide more insights for this issue. *ANTXR2*, also widely known as Capillary morphogenesis gene 2, is a type I membrane protein expressed ubiquitously in human tissues and involved in angiogenesis. Its critical role was well established in diseases beside anthrax, such as in hyaline fibromatosis syndrome, ankylosing spondylitis and potentially in many kinds of cancers [28,29,30]. Given that regulatory activity and CREB binding were cross-validated in different cell types, results obtained from our study might also be indicative for related disease predisposition.

## 4. Experimental Section

### 4.1. Cell Culture

HEK293 cells (ATCC CRL-1573) were maintained in Minimum Essential Medium, Alpha 1X with Earle’s salts, ribonucleotides, deoxyribonucleosides and *L*-glutamine (Cellgro, Manassas, VA, USA) supplemented with 10% fetal bovine serum (ExCell). THP-1 cells (ATCC TIB-202) were maintained in RPMI 1640 Medium (Cellgro) supplemented with 10% fetal bovine serum (ExCell).

### 4.2. Chromosome Conformation Capture Assay

The 3C assay was performed according to Miele *et al.* [31] with some modifications. Following the knowledge gained from Gavrilov *et al.* [32], experiments were conducted in small of volumes (200–500 μL). The nuclear DNA of 1% formaldehyde cross-linked HEK293 cells was digested with EcoRI prior to proximity ligation and the reversal of cross-links. A fragment containing the *ANTXR2* promoter was used as an anchor. DNA fragments that spanned each of the restriction sites to be analyzed were amplified by PCR and equimolecularly mixed as a control template [33]. The interaction frequencies were indicated by the normalized amplification efficiency.

### 4.3. Promoter Activity Assay

Plasmids were introduced to HEK293 cells using Lipofectamine 2000 reagent (Life Technology, Grand Island, NY, USA) as per manufacturer’s protocol. The promoter activity of *ANTXR2* upstream fragments was assessed by Dual-Luciferase^®^ Reporter Assay System (Promega, Madison, WI, USA) following the manufacturer’s instructions.

### 4.4. Fragment Deletion Using CRISPR

Human codon-optimized Cas9 expression plasmid (hCas9) and guide RNA Empty Vector were constructed by Church Lab (Addgene, Cambridge, MA, USA). To delete the putative promoter region of *ANTXR2*, we designed two flanking guide RNAs (5′-GTCATCTTCAACTCGGC-3′ and 5′-GCTCAGGAGGGTCGCAA-3′) and co-transfected cells with hCas9 expression plasmid. The clones were screened using a PCR-based approach [34,35,36,37,38]. The transcripts’ abundance of *ANTXR2* were measured by RT-qPCR.

### 4.5. ChIP-qPCR Assay

ChIP-qPCR assays were performed using UPSTATE One-Day Chromatin Immunoprecipitation Kits with some modifications [39]. HEK293 cells were cross-linked in 1% paraformaldehyde and chromatin sheared to 200–500 bp fragments by sonication. Immune complexes containing CREB were enriched using ChIP-grade anti-CREB antibody (Abcam, Cambridge, MA, USA) and protein-G magnetic beads. After washing under stringent conditions, CREB-bound fragments were eluted in TE buffer with 1% SDS. IgG (Cell Signaling Technology, Danvers, MA, USA) immunoprecipitation was used as a negative control. Primers for promoter region were 5′-AGGTCCTGAGAGGACAAAGGGAGTCT-3′ and 5′-ATTGTCTGCAGGAACTCTCCGGAAT-3′, primers for enhancer region were 5′-CCACTGGTCTGGTGTGACAGTA-3′ and 5′-AGGACTGTAACATAGGGAATCGC-3′.

### 4.6. EMSA

EMSA was performed using LightShift Chemiluminescent EMSA Kit (Pierce Biotechnology, Rockford, IL, USA) following the manufacturer’s instructions. Antibody used in Supershift is ChIP-grade anti-CREB antibody (Abcam, Cambridge, MA, USA).

### 4.7. Statistical Analyses

The experimental data were analyzed by Microsoft Excel 2013 and GraphPad Prism 6.01 (GraphPad Software, Inc., La Jolla, CA, USA). Two samples comparison, including RT-qPCR experiments (Figure 2a,b,e), and association between genotypes and toxin sensitivity (Figure 3d) were analyzed by Student’s *t*-test. Relative promoter activity experiments (Figure 2c right and Figure 3a) and association between genotypes and gene expression were analyzed by one-way ANOVA with Tukey post-test. The error bars in aforementioned figures and Figure 1 bottom represent standard deviation of the samples. *p*-value < 0.05 was considered statistically significant [40].

## 5. Conclusions

Promoter and enhancer elements of *ANTXR2* were identified and two variants, rs13140055 and rs80314910, located in CREB-binding motifs were identified with a combinatorial influence on promoter activity. These two SNPs also contributed to the anthrax toxin sensitivity variation of individuals. These findings further our understanding of human variability in *ANTXR2* expression and anthrax toxin susceptibility.

## Supplementary Materials

The following are available online at www.mdpi.com/2072-6651/8/01/0001/s1.
