# Supplementary material for: Anthrax Susceptibility: Human Genetic Polymorphisms Modulating ANTXR2 Expression
Source: Toxins (Basel). 2015 Dec 22;8(1):1. doi: 10.3390/toxins8010001 (PMC4728523; doi:10.3390/toxins8010001)
Supplement: Supplementary file 1 [file toxins-08-00001-s001.pdf]

Supplementary Materials: Anthrax Susceptibility: Human Genetic Polymorphisms Modulating *ANTXR2* Expression

Zhang Zhang, Yan Zhang, Minglei Shi, Bingyu Ye, Wenlong Shen, Ping Li, Lingyue Xing, Xiaopeng Zhang, Lihua Hou, Junjie Xu, Zhihu Zhao and Wei Chen

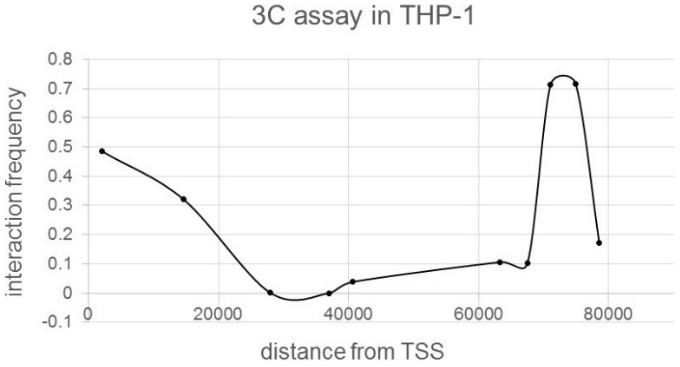

Figure S1. Interaction frequency between fragments digested by restriction enzyme and cognate promoter in cross-linked THP-1 cells.

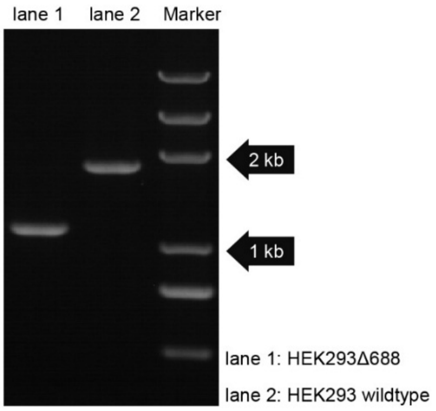

Figure S2. The PCR amplicons of fragment deletion mutant and wildtype.

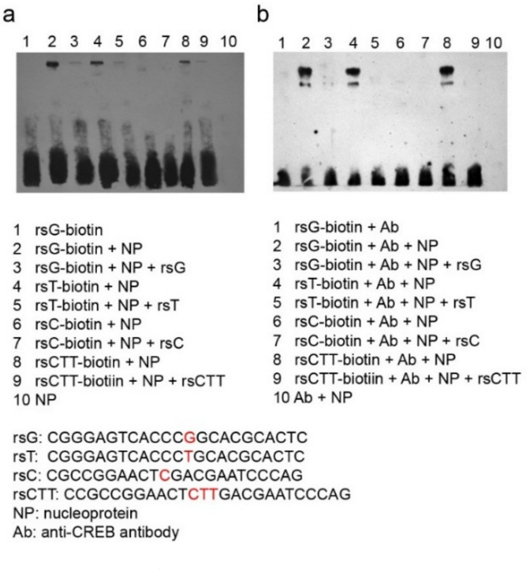

Figure S3. EMSA (**a**) and Supershift (**b**) of nucleoprotein and anti-CREB antibody with probes representing variations of rs13140055 and rs80314910. Information of the lanes were stated below. The red letters highlighted the variations of the SNPs.
